# Coagulation FXIII-A Protein Expression Defines Three Novel Sub-populations in Pediatric B-Cell Progenitor Acute Lymphoblastic Leukemia Characterized by Distinct Gene Expression Signatures

**DOI:** 10.3389/fonc.2019.01063

**Published:** 2019-10-25

**Authors:** Katalin Gyurina, Bettina Kárai, Anikó Ujfalusi, Zsuzsanna Hevessy, Gábor Barna, Pál Jáksó, Gyöngyi Pálfi-Mészáros, Szilárd Póliska, Beáta Scholtz, János Kappelmayer, Gábor Zahuczky, Csongor Kiss

**Affiliations:** ^1^Department of Pediatrics, University of Debrecen, Debrecen, Hungary; ^2^Department of Laboratory of Medicine, University of Debrecen, Debrecen, Hungary; ^3^1st Department of Pathology and Experimental Cancer Research, Semmelweis University, Budapest, Hungary; ^4^Department of Pathology, University of Pécs, Pécs, Hungary; ^5^UD GenoMed Medical Genomic Technologies Ltd., Debrecen, Hungary; ^6^Genomic Medicine and Bioinformatic Core Facility, Department of Biochemistry and Molecular Biology, Faculty of Medicine, University of Debrecen, Debrecen, Hungary

**Keywords:** pediatric BCP-ALL, FXIII-A, *F13A1*, gene expression signature, B-other genotype, oligonucleotide microarray, RT-Q-PCR

## Abstract

**Background:** Leukemic B-cell precursor (BCP) lymphoblasts were identified as a novel expression site for coagulation factor XIII subunit A (FXIII-A). Flow cytometry (FC) revealed three distinct expression patterns, i.e., FXIII-A negative, FXIII-A dim, and FXIII-A bright subgroups. The FXIII-A negative subgroup was significantly associated with the “B-other” genetic category and had an unfavorable disease outcome.

**Methods:** RNA was extracted from bone marrow lymphoblasts of 42 pediatric patients with BCP-acute lymphoblastic leukemia (ALL). FXIII-A expression was determined by multiparameter FC. Genetic diagnosis was based on conventional cytogenetic method and fluorescence *in situ* hybridization. Affymetrix GeneChip Human Primeview array was used to analyze global expression pattern of 28,869 well-annotated genes. Microarray data were analyzed by Genespring GX14.9.1 software. Gene Ontology analysis was performed using Cytoscape 3.4.0 software with ClueGO application. Selected differentially expressed genes were validated by RT-Q-PCR.

**Results:** We demonstrated, for the first time, the general expression of *F13A1* gene in pediatric BCP-ALL samples. The intensity of *F13A1* expression corresponded to the FXIII-A protein expression subgroups which defined three characteristic and distinct gene expression signatures detected by Affymetrix oligonucleotide microarrays. Relative gene expression intensity of *ANGPTL2, EHMT1 FOXO1, HAP1, NUCKS1, NUP43, PIK3CG, RAPGEF5, SEMA6A, SPIN1, TRH*, and *WASF2* followed the pattern of change in the intensity of the expression of the *F13A1* gene. Common enhancer elements of these genes revealed by *in silico* analysis suggest that common transcription factors may regulate the expression of these genes in a similar fashion. *PLAC8* was downregulated in the FXIII-A bright subgroup. Gene expression signature of the FXIII-A negative subgroup showed an overlap with the signature of “B-other” samples. *DFFA, GIGYF1, GIGYF2*, and *INTS3* were upregulated and *CD3G* was downregulated in the “B-other” subgroup. Validated genes proved biologically and clinically relevant. We described differential expression of genes not shown previously to be associated with pediatric BCP-ALL.

**Conclusions:** Gene expression signature according to FXIII-A protein expression status defined three novel subgroups of pediatric BCP-ALL. Multiparameter FC appears to be an easy-to-use and affordable method to help in selecting FXIII-A negative patients who require a more elaborate and expensive molecular genetic investigation to design precision treatment.

## Introduction

According to current knowledge, acute lymphoblastic leukemia (ALL) can be best characterized by an integrated set of clinical, pathological, morphologic, immunophenotypic, and genetic properties. Detailed characterization of the neoplastic clones of children with ALL allowed the development of highly effective risk-tailored therapies. Introduction of advanced diagnostic tools helped in identifying an increasing number of molecular targets and may contribute to the application of personalized treatment offering cure for each individual patient. Recurrent genetic aberrations represent the basis of the classification of ALL according to the 4th edition of the WHO Classification of Tumors of Hematopoietic and Lymphoid Tissues in 2008, and its 2016 revision (1, 2). Gene expression profiling by oligonucleotide microarray was shown to contribute to conventional and molecular cytogenetics by improving diagnostic accuracy and prognostic relevance as well as by defining new entities. The “B-other” genetic subgroup, i.e., BCP-ALL cases without established recurrent genetic abnormalities exhibits alterations to be revealed by advanced genomic technologies. Within “B-other” ALL, Philadelphia-like (Ph-like) or *BCR-ABL1*-like ALL, a provisional entity of the 2016 revision of the WHO classification, has been defined based on gene expression signature similar to Ph-positive/*BCR-ABL1*-positive ALL and characteristically distinct from the rest of BCP-ALL cases (“non-B-other”) (3–6). Recently, fourteen B-cell precursor (BCP)-ALL subgroups were defined by analyzing a large sample set of an international study using RNA-sequencing. The study revealed six new subgroups in addition to previously identified ones demonstrating the diagnostic utility and prognostic power of gene expression studies in pediatric BCP-ALL (7).

Our group identified BCP-ALL blasts as a new expression site for coagulation factor XIII subunit A (FXIII-A). In contrast to the BCP-ALL blasts, neither normal bone marrow B-cell progenitors, nor mature B-cells, both normal and leukemic once, nor T-lymphoblasts/lymphocytes express FXIII-A (8). We observed three characteristic expression patterns by flow cytometry (FC): FXIII-A negative, FXIII-A dim, and FXIII-A bright lymphoblasts with about two thirds of pediatric BCP-ALL patients representing FXIII-A positive (FXIII-A dim and bright) cases. According to our retrospective clinical investigation, patients with FXIII-A negative lymphoblasts had significantly worse event-free and overall survival than patients with FXIII-A positive lymphoblasts. Moreover, the “B-other” genetic subtype was significantly more frequent within the FXIII-A negative than in the FXIII-A positive subgroup (9). The prognostic importance of FXIII-A expression by FC was confirmed and further specified in a recently concluded prospective study suggesting a favorable prognostic effect of the FXIII-A dim expression pattern within the FXIII-A positive subgroup (unpublished results). Here we present results, for the first time, on gene expression signatures associated with the three characteristic FXIII-A protein expression patterns. In addition, we investigated overlaps between the gene expression profile of the FXIII-A negative protein expression subgroup with the gene expression profile of the “B-other” subgroup.

## Materials and Methods

### Patients

Samples were collected from pediatric patients with BCP-ALL treated in four Hungarian Pediatric Hematology and Oncology Departments (University of Debrecen, Debrecen; Semmelweis University, Budapest; University of Pécs, Pécs; BAZ Country Hospital and University Teaching Hospital, Miskolc) between September 1, 2015 and August 31, 2018. Patients were treated according to BFM ALL-IC 2009 clinical trial (EuDraCT No: 2010-019722-13). Patients with *BCR-ABL1* rearrangement [*t*_(9, 22)_] were excluded, since they were not treated according to BFM ALL-IC 2009 protocol. Quantitatively and qualitatively suitable RNA was separated from 42 patients with FXIII-A negative (14), FXIII-A dim (21), and FXIII-A bright (7) BCP-ALL.

Excess bone marrow (BM) samples were collected after signed informed consent obtained from study participants and their legal caregivers. The study was approved by the Scientific Research Ethical Committee (“TUKEB”) of the Medical Research Council of Hungary: No. 43033-1/2014/EUK(423/2014) and was carried out according to the Code of Ethics of the World Medical Association and the ethical standards of the 2000 Revision of the Helsinki Declaration.

### Immunophenotype Analysis

The FXIII-A expression pattern was determined by multiparameter FC analysis as published before (9). Bone marrow samples were examined for the following antibody combination: cytoplasmic FXIII-A(FITC)–CD10(PE)–CD45(PerCP-Cy5.5)–CD19(APC)–CD19(PECY7). CD19, CD45 markers were purchased from Becton Dickinson Biosciences (San Jose, CA, USA), CD10 marker were purchased from DAKO (Glostrup, Denmark), CD19(PECY7) marker were purchased from Sony Biotechnology (San Jose, CA, USA). Generation and fluorescent isotihocyanate (FITC) labeling of mouse monoclonal antibody against FXIII-A was carried out as previously described (10). One-hundred thousand events were acquired with the help of FacsCanto-II (Becton Dickinson Biosciences, San Jose, CA, USA) and Navios (Beckman Coulter, Brea, CA, USA) flow cytometers. According to the intensity of FXIII-A expression, patients were assigned to three groups: BCP-ALL with FXIII-A negative blasts (<20% FXIII-A positive lymphoblasts; FXIII-A negative group), BCP-ALL with moderate FXIII-A expression (20–79% FXIII-A positive lymphoblasts; FXIII-A dim group), and BCP-ALL with strong FXIII-A expression (≥80% FXIII-A positive lymphoblasts; FXIII-A bright group). To determine FXIII-A expression of leukemic cells, normal residual lymphocytes served as a negative control.

FC data were analyzed by FACSDiva (Becton Dickinson, Franklin Lakes, NJ, USA) or Kaluza (Beckman Coulter, Brea, CA, USA) softwares. Flow cytometers were subjected to daily performance checks, using Cytometer Setup and Tracking (Becton Dickinson, Franklin Lakes, NJ, USA) or Flow Check Pro (Beckman Coulter, Brea, CA, USA) fluorescent microbeads.

### Genetic Investigations

Cytogenetic analysis was performed on unstimulated 24 h cultures of BM according to standard protocol. Fluorescence *in situ* hybridization (FISH) was carried out on cells from the same BM samples using commercially available probe sets (*BCR-ABL, ETV6-RUNX1, KMT2A)*. Patients with *t*_(12, 21)_/*ETV6/RUNX1* or high hyperdiploidy (51–65 chromosome number) were considered as low-risk group. The high-risk group consisted of patients with *KMT2A* rearrangements, iAMP21, complex karyotype, near haploidy (chromosome number 23–29), and low hypodiploidy (chromosome number <45). Patients with *t*_(1, 19)_, and all other genetic subgroups not fitting in the low- and high-risk categories, including the “B-other” subgroup were considered as intermediate-risk group (6, 11).

### RNA Preparation

Excess BM samples from BCP-ALL patients were collected into PAXgene Blood RNA Plastic Tube (PreAnaltyX, Hombrechtikon, Switzerland). Total cellular RNA was isolated using PAXgene Blood miRNA Kit (PreAnalityX) according to conventional protocol. Qualitative and quantitative analyses of RNA were performed using Agilent Bioanalyzer (Agilent Technologies, La Jolla, CA, USA). Samples with an RNA Integrity number above 8.0 were used in MicroArray analysis.

### Microarray Analysis

Affymetrix GeneChip Human Primeview array (Affymetrix, Santa Clara, CA, USA) was used to analyze global expression pattern of 28,869 well-annotated genes. 3'IVT Expression Kit (Affymetrix) and GeneChip WT Terminal Labeling and Control Kit (Affymetrix) were used for amplifying and labeling 250 ng of RNA samples. Samples were hybridized at 45°C for 16 h and then standard washing protocol was performed using GeneChip Fluidics Station 450 (Affymetrix) and the arrays were scanned on GeneChip Scanner 7G (Affymetrix) procedure. Data of this study have been deposited in NCBI's Gene Expression Omnibus and are accessible through GEO Series accession number GSE134480 (https://www.ncbi.nlm.nih.gov/geo/query/acc.cgi?acc=GSE134480).

### Gene Ontology Analysis

Gene Ontology (GO) analysis was performed using Cytoscape 3.4.0 software (cytoscape.org) with ClueGO application. The settings were the following: GO biological process and GO immune system process. For statistical analysis two-sided hypergeometric test and Benjamini-Hochberg FDR were used. Significantly enriched GO categories were considered to *p*-value <0.05 and κ score <0.4.

### Real Time Quantitative Polymerase Chain Reaction (RT-Q-PCR) Validation of Microarray Data

For RT-Q-PCR validation of microarray data pre-designed, factory-loaded 384-well TaqMan low-density array (ThermoFisher Scientific, Waltham, MA, USA) was used to determine the level of expression of selected genes using technical duplicates. Genes for validation were selected based on fold-changes (with fold-changes >2.0 cut-off) between any of the pre-defined subgroups and based on putative biological and clinical relevance of gene functions as defined by GO analysis data (Table 1). RT-Q-PCR expression levels of target genes were normalized to the mean of *B2M, GAPDH*, and *GUSB* reference genes. Normalized gene expression values were calculated based on the ΔC_t_ method, where relative expression equals 2^−ΔCt^, where ΔC_t_ represents the threshold cycle (C_t_) of the target minus that of the mean of reference genes.

**Table 1:** Genes selected for validation by RT-Q-PCR based either on gene expression fold-changes detected by Affymetrix Microarray (in bold characters) or based on selected GO annotations.

**Genes selected for validation**	**Fold changes detected by Affymetrix MicroArray**			**GO annotations**
	**FXIII-A negative/FXIII-A dim**	**FXIII-A negative/FXIII-A bright**	**FXIII-A bright/FXIII-A dim**	
**WASF2**	**0.94**	**0.50**	**0.53**	**Angiogenesis**
BCL2L1	0.79	0.60	0.75	Apoptosis regulation
CASP2	0.90	0.79	0.88	Apoptosis regulation
DFFA	0.33	0.39	1.19	Apoptosis regulation
PAK2	1.91	1.07	0.56	Apoptosis regulation
PIK3CG	1.58	1.62	1.03	Apoptosis regulation
PKN2	1.43	1.26	0.88	Apoptosis regulation
**SEMA6A**	**1.06**	**2.38**	**2.23**	**Apoptotic process**
**CLSTN1**	**0.51**	**0.45**	**0.88**	**Calcium ion binding**
IL7R	1.03	0.98	0.94	Cell differentiation
PLAC8	1.30	1.33	1.02	Cell differentiation
RORA	0.92	1.01	1.10	Cell differentiation
**NUCKS1**	**1.56**	**1.41**	**0.90**	**Cell differentiation**
**TRH**	**2.05**	**1.64**	**0.80**	**Cell-cell signaling**
FOXO1	1.53	1.28	0.84	Cellular response to glucocorticoid stimulus
CX3CR1	0.83	1.63	1.97	Chemokine receptor activity
EHMT1^*^	1.18	1.22	1.03	Chromatin modification
ING5^*^	0.97	1.25	1.29	Chromatin modification
JMJD1C	0.97	0.67	0.69	Chromatin modification
WAC	1.24	1.50	1.21	Chromatin modification
SART3	5.90	2.55	0.43	Chromatin modification
MDM2^*^	**1.28**	**1.14**	**0.89**	**Identical protein binding**
GIGYF1	3.84	5.07	1.32	Insulin-like growth factor receptor signaling pathway
GIGYF2	1.08	1.06	0.98	Insulin-like growth factor receptor signaling pathway
**TAOK1**	**4.60**	**2.67**	**0.58**	**Kinase activity**
**SPIN1**	**1.28**	**1.27**	**0.99**	**Methylated histone binding**
**MAP4**	**1.29**	**1.19**	**0.92**	**Microtubule binding**
AKAP13	1.14	1.16	1.02	Nuclear export
KHDRBS1	1.66	1.21	0.73	Nuclear export
MAGOH	1.35	1.25	0.92	Nuclear export
NUP43^*^	1.12	1.00	0.89	Nuclear export
**ZC3H11A**	**12.64**	**3.37**	**0.27**	**Nuclear export**
POLDIP3	1.60	1.48	0.92	Nuclear export
SRSF5	1.14	1.28	1.12	Nuclear export
FGFR1OP	7.20	2.50	0.35	Nuclear export
HAP1	1.07	0.86	0.80	Post-transcriptional regulation of gene expression
**F13A1**	**1.86**	**1.60**	**0.86**	**Protein-glutamine gamma-glutamyltransferase activity**
CCL5	1.16	1.12	0.97	Protein homodimerization
CD3G	0.80	0.65	0.81	Protein heterodimerization
**RAPGEF5**	**1.35**	**1.22**	**0.90**	**Ras signaling pathway**
**ANGPTL2**	**16.94**	**2.89**	**0.17**	**Signaling receptor binding**
DHX36	0.83	0.80	0.96	Translation regulation
INTS3	17.71	3.41	0.19	Translation regulation
RC3H1	0.69	0.46	0.67	Translation regulation
SECISBP2L	1.45	1.54	1.06	Translation regulation

*For definition of the three different FXIII-A expression groups see text*.

Genes marked in ^*^ have also been listed under the GO term: peptidyl-lysine modification (GO:0018205).

### *In silico* Investigation of Validated DE Genes

Interactions of validated genes and *F13A1* gene were investigated using STRING v11. (12) and GeneHancer (13) databases. STRING v11 database contains putative protein-protein interactions predicted on a well-defined score system. GeneHancer portrays 285 000 integrated candidate enhancers and subsequently links enhancers to genes.

### Statistical Analysis

Microarray data were analyzed by Genespring GX14.9.1 software (Agilent Technologies, La Jolla, CA, USA). To identify statistically significant genes, we used volcano plot analysis. The resulting scatterplot showed statistical significance (*p*-value) vs. magnitude of change (fold-change). Affymetrix data files were imported using the Robust Multi-Array Average algorithm and median normalization was performed. To identify differentially expressed genes between pre-defined data sets, statistical analysis was performed using ANOVA with Tukey *post-hoc* test (14) and moderated *T*-test, Benjamini-Hochberg False Discovery Rate was used for multiple testing corrections, *p*-value <0.05 was considered as significant difference.

## Results

### Characterization of Samples of Children With BCP-ALL

**Figure 1:**
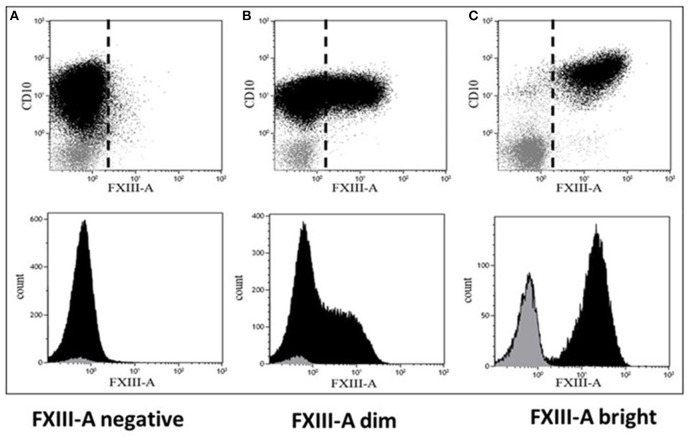
Representative dot plots and histograms of leukemic lymphoblasts. There are three different patterns of cytoplasmic FXIII-A expression in terms of positivity of leukemic lymphoblasts: **(A)** negative expression pattern below 20%, **(B)** dim expression pattern between 20 and 80%, and **(C)** bright expression pattern ≥80% of leukemic lymphoblasts (black) with a FXIII-A staining exceeding the intensity of negative controls, i.e., residual normal lymphocytes (gray). Based on FXIII-A expression intensity of normal residual lymphocytes, the threshold of positivity is marked by the dashed line on the respective dot-plots (upper quadrants). The intensity of the FXIII-A expression increased continuously, as the histogram of lymphoblasts with dim expression pattern shows (**B**, lower quadrant), which excludes the existence of a distinct FXIII-A negative and a FXIII-A bright sub-population.

RNA samples obtained from 42 pediatric patients with BCP-ALL were used for gene expression profiling investigations (Table 2). Cytoplasmic expression pattern of FXIII-A by FC successfully divided patients into three categories: patients with FXIII-A negative, FXIII-A dim and FXIII-A bright BCP-ALL. Representative FC dot plots and histograms are shown in Figure 1. Regarding genetic categories, 27 patients had recurrent genetic abnormalities and 15 patients were assigned to the “B-other” subgroup: 7/12 FXIII-A negative patients, 6/21 FXIII-A dim patients, and 2/7 FXIII-A bright patients.

**Table 2:** Clinical and pathological characterization of patiens with BCP-ALL.

**Patient ID code**	**Sex**	**Age (years)**	**Initial WBC (G/L)**	**Ratio of FXIII-A positive cells (%)**	**Genetic category**	**Outcome (dead/alive)**
cALL_RNS_13	M	8	3.9	10.9	iAMP21	Dead
cALL_RNS_14	M	11	19	1.4	B-other	Dead
cALL_RNS_15	M	3	5.9	5.1	B-other	Alive
cALL_RNS_16	M	2	23.38	0.9	Hyperdiploid	Dead
cALL_RNS_17	F	4	25.9	1.7	B-other	Alive
cALL_RNS_26	M	0	8.33	2	Hyperdiploid	Alive
cALL_RNS_33	F	12	22.6	5	B-other	Alive
cALL_RNS_34	F	13	98.8	13	B-other	Alive
cALL_RNS_35	M	2	10.45	1.8	ETV6/RUNX1	Alive
cALL_RNS_36	F	1	11.03	4	TCF3/PBX1	Alive
cALL_RNS_53	M	2	5.51	11.4	B-other	Dead
cALL_RNS_54	F	3	18.33	8.4	B-other	Dead
cALL_RNS_18	M	2	5.9	1.1	Hyperdiploid	Alive
cALL_RNS_19	M	3	15.9	10.6	KMT2A	Alive
cALL_RNS_45	F	1	11.37	28.8	B-other	Dead
cALL_RNS_46	F	1	5.71	32.6	ETV6/RUNX1	Dead
cALL_RNS_47	M	0	377	23	KMT2A	Alive
cALL_RNS_58	F	4	12.14	41	Hyperdiploid	Alive
cALL_RNS_59	F	3	4.4	48.9	Hyperdiploid	Alive
cALL_RNS_60	M	6	3.31	34.6	Hyperdiploid	Alive
cALL_RNS_61	M	4	8.9	52	ETV6/RUNX1	Alive
cALL_RNS_62	F	2	21.91	71.9	Hyperdiploid	Alive
cALL_RNS_63	M	3	3.62	45	ETV6/RUNX1	Alive
cALL_RNS_64	F	2	5.3	71.9	ETV6/RUNX1	Alive
cALL_RNS_44	M	8	19.73	76.8	Hyperdiploid	Alive
cALL_RNS_50	M	3	7.28	59.6	B-other	Alive
cALL_RNS_52	F	3	83.69	58	B-other	Alive
cALL_RNS_55	F	2	12.34	56	Hyperdiploid	Dead
cALL_RNS_56	M	12	10.5	24.9	B-other	Dead
cALL_RNS_57	F	7	1.33	35.6	Hyperdiploid	Alive
cALL_RNS_51	F	16	12.38	25	B-other	Alive
cALL_RNS_49	M	2	14.5	22.4	B-other	Alive
cALL_RNS_02	F	10	6.51	95	B-other	Dead
cALL_RNS_03	F	15	5.64	72.5	Hyperdiploid	Alive
cALL_RNS_04	F	5	5.1	69	ETV6/RUNX1	Alive
cALL_RNS_01	F	1	222.8	85	B-other	Dead
cALL_RNS_05	F	12	19.54	95	ETV6/RUNX1	Alive
cALL_RNS_06	F	16	48.55	94	KMT2A	Alive
cALL_RNS_07	F	5	61.6	88	ETV6/RUNX1	Alive
cALL_RNS_08	F	2	2.72	91	Hyperdiploid	Alive
cALL_RNS_09	F	4	8.12	82.3	ETV6/RUNX1	Alive
cALL_RNS_37	M	16	20.1	98.9	Hyperdiploid	Alive

### Gene Expression Profiles of BCP-ALL Samples

**Figure 2:**
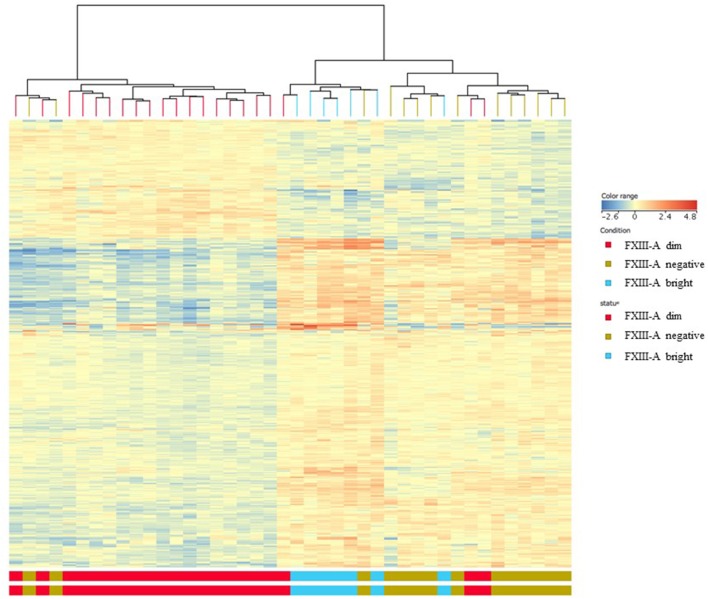
Gene expression signatures according to FXIII-A protein expression; heat map analysis. With the exception of one to two outliers within the respective groups, heat map analysis exhibited three different patterns of gene expression for each, FXIII-A negative (green color), FXIII-A dim (red color), and FXIII-A bright (blue color) subgroups.

Differentially expressed (DE) genes were identified using two distinctive features: FXIII-A protein expression determined by FC and “B-other” status. DE genes were screened by volcano plot filtering. There were 26 genes found when comparing the FXIII-A negative with the FXIII-A bright subgroup. The FXIII-A dim vs. bright comparison resulted in 155 DE genes and there were 88 DE genes identified between the FXIII-A negative and dim subgroups. With the exception of one to two outliers within the respective groups, heat map analysis exhibited three different patterns of gene expression for each, FXIII-A negative, FXIII-A dim, and FXIII-A bright subgroup. Importantly, FXIII-A negative and bright samples clustered close together and were well-separated from the FXIII-A dim subgroup (Figure 2).

**Figure 3:**
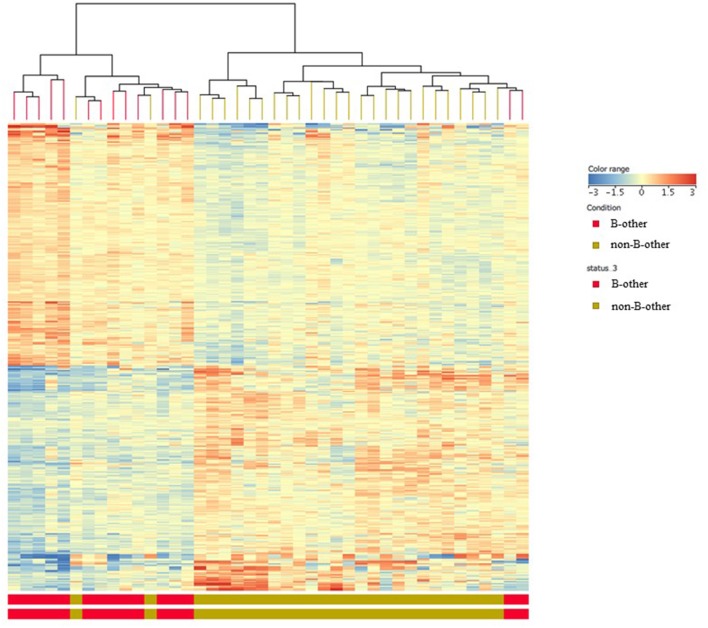
Gene expression signatures according to “B-other” status; heat map analysis. Heat map analysis clearly showed two distinct sub-populations: gene expression signature of the “B-other” subgroup (red color) differed characteristically from the gene expression signature of the rest of samples, the “non-B-other” subgroup (green color).

**Figure 4:**
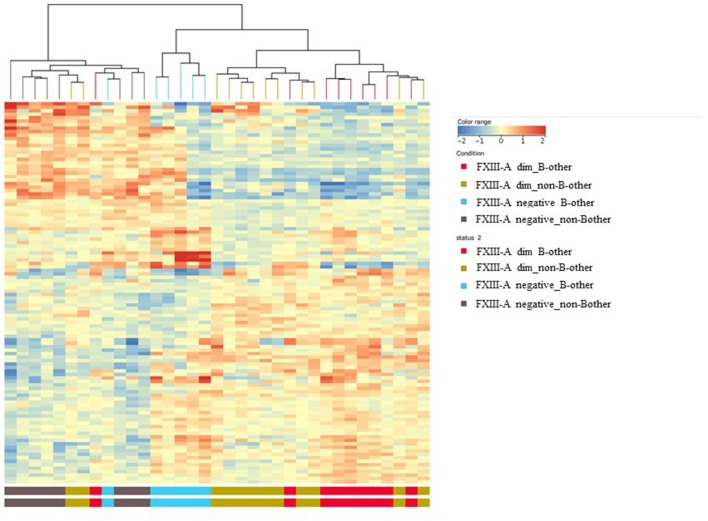
Overlap between the gene expression signatures of FXIII-A negative and “B-other” samples; heat map analysis. Heat map analysis confirmed that, except for two outliers, gene expression profile characterizing FXIII-A negative samples overlapped with gene expression profile characterizing samples of the “B-other” subgroup.

Comparing the gene expression signature of the “B-other” subgroup with the rest of patient samples, the so called “non-B-other” group, 142 DE genes were found after filtering to 1.5-fold-change. Heat map analysis clearly showed two distinct sub-populations: gene expression signature of the “B-other” subgroup differed characteristically from the gene expression signature of the “non-B-other” subgroup (Figure 3). Then we investigated how DE genes, characteristic for “B-other” status, were related to DE genes of subgroups according to FXIII-A expression pattern. We found 32 DE genes expressing exclusively in the FXIII-A negative and “B-other” group vs. FXIII-A negative and the “non-B-other” group. Heat map analysis confirmed that, with the exception of one outlier, gene expression profile characterizing FXIII-A negative samples overlapped with gene expression profile characterizing samples of the “B-other” subgroup (Figure 4).

### Functional Characterization of Differentially Expressed Genes in the BCP-ALL Subgroups

Identification of enriched functional categories according to the FXIII-A protein expression pattern, DE genes were categorized into 156 GO processes. Nevertheless, most of them, in particular those with the strongest statistical *p*-values, were related to epigenetic and/or gene expression regulatory processes such as histone modification, chromatin organization, RNA destabilization, post-transcriptional regulation of gene expression, etc., or other regulatory and cellular processes, such as apoptosis and morphogenesis (Supplementary Table 1). In addition, we identified biological processes resulting in peptidyl-lysine modification, which are in relation with the known physiological function of FXIII-A catalyzing the formation of γ-glutamyl-ε-lysyl amide crosslinks between fibrin monomers to form an insoluble clot (Supplementary Table 1).

In the “B-other” status comparison of DE genes with a fold-change >2.0, much less GO processes were identified than according to the FXIII-A status comparison. Genes involved in lymphocyte and T-cell apoptotic processes (*CCL5, CD3G, IL7R, PLAC8*) were over-presented, as well as two others, *CX3CR1, RORA*, corresponding to macrophage migration. When decreasing the filtering threshold to fold-change >1.5, additional biological processes proved significant represented by the following genes, *BCL10, CX3CR1, GNLY, PTPRC, STK4, TNFSF10, ATP2B1, DNAJC3, PLAC8, THRA, MAPKBP1, PER1, RORA, USP32, CCL5, GNG2, PLCB3, BCL10, CCL5, CD3G, IL7R* (Supplementary Table 2).

### Validation of Global Transcriptomics Data

From the oligonucleotide microarray results of DE genes, either according to FXIII-A expression status or according to “B-other” genetic status we selected 45 genes for validation by RT-Q-PCR. Selection of 13/45 genes was based on fold change results, whereas an additional 32/45 genes were selected according to enriched functional categories of potential interest as defined by the GO analysis (Table 1). We were not able to detect transcripts of *RORA* by RT-Q-PCR which might have a technical reason.

### FXIII-A Expression-Based Results

**Figure 5:**
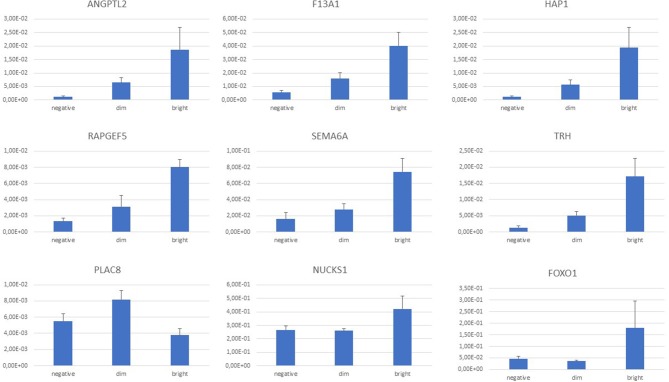
Normalized gene expression values by RT-Q-PCR according to FXIII-A protein expression status; graph diagram. There was a continuous increase in normalized gene expression levels from FXIII-A negative through dim to bright subgroups that was endogenously validated by the *F13A1* differential expression within the three FXIII-A protein expression groups. *ANGPTL2, NUCKS1, RAPGEF5*, and *SEMA6A* followed this trend. Based on the intensity of the differential expression, separation of *FOXO1, HAP1*, and *TRH* genes of the FXIII-A bright subgroup were more prominent. *PLAC8* expression was most intensive in the FXIII-A dim subgroup.

Expression of *F13A1* gene was detected and readily validated by RT-Q-PCR in every sample. Intensity of gene expression; however, was characteristically different among samples of the three different FXIII-A protein expression subgroups with an increasing intensity in terms of relative fold-changes measured by RT-Q-PCR from the FXIII-A negative, through dim to bright subgroups (Figure 5).

Similarly, most of the genes (8/13 *p* < 0.05, 9/13 *p* < 0.10) from the group selected on the basis of highest fold changes between any two groups among FXIII-A negative, dim and bright groups according to the microarray results were validated by RT-Q-PCR. Of the 25 genes selected on basis of functional significance according to GO analysis, four genes could be validated. Within the GO group of translation regulation there were 2/6 with *p* < 0.05, and 3/6 with *p* < 0.10 (Table 3) genes that could be validated, providing a considerably better ratio than it was found in the subgroups of apoptosis regulation (0/3), chromatin modification (1/5), and nuclear export (0/5) (Tables 1, 3). Generally, fold-changes were enhanced in RT-Q-PCR compared to microarray data (Supplementary Table 3). *PLAC8* and *HAP1* represent impressive examples. DE of *PLAC8*, a placenta-specific gene of unrevealed function, was not found significant by microarray but turned out significant upon validation. *HAP1*, a member of the translation regulation GO group, exhibited a very slight overexpression by microarray and it proved much stronger by RT-Q-PCR.

**Table 3:** Relative expression, enriched functional categories according to GO annotations and clinical relevance of validated genes accordig to FXIII-A protein expression patterns.

**Gene**	**Normalized fold-change by RT-Q-PCR**				**GO annotations**	**Clinical relevance in leukemia and cancer**	**References**
	*P*-value^*^	**FXIII-A negative**	**FXIII-A dim**	**FXIII-A bright**			
*ANGPTL2*	0.0061	1.10E-03	6.47E-03	1.87E-02	Signaling receptor binding	*ETV6* target gene in pediatric ALL	(15)
*EHMT1*	0.0401	4.79E-02	5.55E-02	8.89E-02	Chromatin modification	Transcriptional coactivator involved in glucocorticoid-induced cell death	(16)
*F13A1*	0.0013	5.58E-03	1.61E-02	4.02E-02	Protein-glutamine gamma-glutamzltransferase activitz	See article	
*FOXO1*	0.0371	4.69E-02	3.56E-02	1.80E-01	Cellular response to glucocorticoid stimulus	Key gene of the *AKT/FOXO1* pathway involved in apoptosis regulation of ALL blasts	(17–19)
*HAP1*	0.0016	1.10E-03	5.69E-03	1.94E-02	Post-transcriptional regulation of gene expression	Negative asparaginase-resistance biomarker in ALL	(20)
*NUCKS1*	0.0182	2.67E-01	2.59E-01	4.21E-01	Cell differentation	Downregulated in VCR-resistant ALL blasts	(21)
*NUP43*	0.0424	1.03E-02	1.17E-02	1.47E-02	Nuclear export	W/o proven function in cancer	
*PIK3CG*	0.0037	1.15E-01	1.24E-01	1.84E-01	Apoptosis regulation	*PIK3/AKT* pathwayplays key regulatory role in BCP-ALL	(22, 23)
*PLAC8*	0.0362	5.51E-03	8.20E-03	3.79E-03	Cell differentiation	Gene of unknown function, activated in various types of mammalian and human cancer	(24, 25)
*RAPGEF5*	0.0192	1.36E-03	3.14E-03	8.01E-03	RAS signal pathway	Abnormal expression involved in papillary thyroid cancer	(26)
*SEMA6A*	0.0019	1.61E-02	2.78E-02	7.43E-02	Apoptotic process	Overexpressed in *ETV6/RUNX1* ALL	(27)
*SPIN1*	0.0077	1.35E-01	1.68E-01	2.76E-01	Chromatin organization	Downregulation induces p53 activation in human cancer cells	(28)
TRH	0.0002	1.36E-03	5.09E-03	1.72E-02	Cell-cell signaling	Deregulation implicated in breast cancer	(29)
WASF2	0.0002	1.11E-01	1.55E-01	2.95E-01	Angiogenesis	Recurrent *WASF2/FGR* fusions are involved in suamous cell cancer, cystadenocarcinoma, and melanoma	(30)

* *P-value of annova analysis comparing the FXIII-A negative, FXIII-A dim, FXIII-A bright groups*.

Considering individual normalized gene expression values, in most of the cases, there was a clear trend of a continuous increase from FXIII-A negative through dim to bright subgroups that was endogenously validated by the *F13A1* relative expression. *ANGPTL2, NUCKS1, RAPGEF5*, and *SEMA6A* followed this trend (Figure 5). Relative fold-changes were similar whether determined by microarray measurements or RT-Q-PCR. In case of *FOXO1, HAP1*, and *TRH*, separation of the bright subgroup from the other two subgroups seemed more prominent and showed a relatively higher fold-change value as determined by RT-Q-PCR than compared to the microarray results. With the exception of *PLAC8*, validated DE genes were downregulated in the FXIII-A negative subgroup compared to the two other subgroups (Supplementary Table 3).

Potential interactions could not be revealed between the protein products of validated genes and FXIII-A using STRING v11 functional protein association networks database. Nevertheless, we could identify a FXIII-A dependent expression of *FOXO1*. Using GeneHancer database we could *in silico* identify enhancers that might have roles in the parallel upregulation of *F13A1* and validated genes. Transcription factor binding sites of *ATF7, POLR2A, RAD21, SMARCA5* gene products were identified for all of the 14 genes shown in Table 3.

## “B-other” Status-Based Results

**Figure 6:**
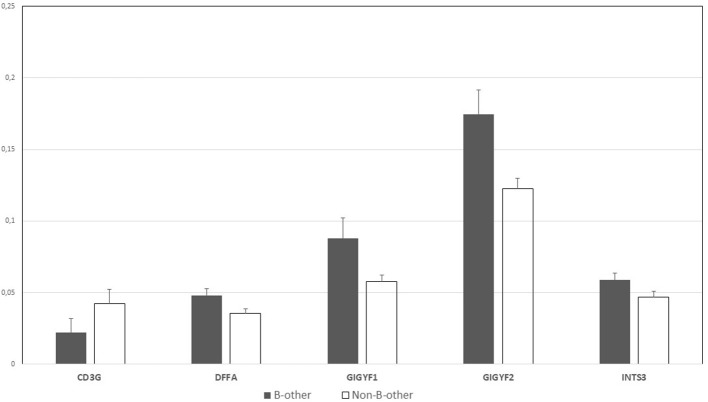
Normalized gene expression values by RT-Q-PCR according to the “B-other” status; graph diagram. *DFFA, GIGYF1, GIGYF2* and *INTS3* genes were overexpressed in “B-other” vs. “non-B-other” samples and in turn, *CD3G* exhibited a relatively lower expression in the “B-other” vs. “non-B-other” samples.

For the “B-other” status-based validation, DE genes related to biological processes (macrophage migration, lymphocyte apoptotic process, T cell apoptotic process, and their regulation), were selected for validation, as GO analysis results were less diverse than in the case of FXIII-A expression-based GO annotations (Supplementary Table 1). We were able to validate the differential expression of five genes by RT-Q-PCR. *DFFA, GIGYF1, GIGYF2*, and *INTS3* were overexpressed in “B-other” vs. “non-B-other” samples and in turn, *CD3G* exhibited a relatively lower expression in the “B-other” vs. “non-B-other” samples (Figure 6). *RORA, IL7R, CCL5, PLAC8, CX3CR* genes, selected for validation based on their functions and fold-changes detected by microarray, could not be validated.

## Discussion

FXIII-A is a useful marker of acute myeloid leukemia (AML) of the monocyte and megakaryocyte lineages (31–34). However, its expression in BCP-ALL blasts was an unexpected finding, since, in contrast to the normal myeloid counterparts of AML blasts, normal B-lymphocytes and their precursors do not express FXIII-A (8). Intracellular expression of FXIII-A protein can be consistently demonstrated in about two thirds of pediatric BCP-ALL samples by FC. Interestingly, the FXIII-A negative status was shown to be significantly associated with the “B-other” genetic subtype and patients with FXIII-A negative BCP-ALL had a significantly worse disease outcome than patients with FXIII-A positive lymphoblasts (9). These facts together suggested that pathological expression of FXIII-A in leukemic BCP blasts may define one or more sub-populations according to the FXIII-A expression pattern by FC.

Little is known on the transcriptional regulation of the *F13A1* gene. Molecular investigations of myeloid leukemia cell lines revealed the presence of binding sites for ubiquitous (NF-1 and SP-1) and myeloid enriched (MZF-1-like protein, GATA-1, and Ets-1) transcription factors in the promoter region of the gene (35). Interestingly, transcription factor binding sites for SP-1, GATA-1, and Ets-1 could not be confirmed by the ENCODE ChIP-seq datasets (data not shown) (36). A more recent study demonstrated that FXIII-A was synergistically regulated by IL-4 and dexamethasone in alternatively activated macrophages and these regulatory molecules are relevant for both normal and leukemic BCPs as well (37). However, transcriptional regulation of the *F13A1* gene in leukemic lymphoblasts has not yet been studied.

Therefore, we decided to investigate the gene expression profile of BCP lymphoblasts according to their FXIII-A expression status. Preliminary results of an ongoing prospective, multi-centric clinical study performed by our group suggested the clinical relevance of the sub-populations characterized by the FXIII-A expression pattern, since patients with FXIII-A dim but not bright lymphoblasts had a better disease outcome than patients with FXIII-A negative lymphoblasts (unpublished preliminary results). Accordingly, we investigated the global gene expression signature of three sample sets.

We have revealed that the three groups according to FXIII-A expression pattern had unique gene expression signatures which were characteristically different from each other. GO analysis of data resulted in a number of biologically relevant enriched functional categories. Identification of biological processes resulting in peptidyl-lysine modification supported the clinical relevance of our findings. FXIII-A is an enzyme catalyzing the formation of γ-glutamyl-ε-lysyl amide crosslinks. In addition to its well-known role in the formation of stable fibrin clot, FXIII-A participates also in much less characterized intracellular regulatory processes including differentiation of monocyte/macrophages, osteoblast, and osteoclast (38). In platelets, FXIII-A has been shown to crosslink cytoskeletal proteins (39). Should FXIII-A participate in similar processes in leukemic BCP lymphoblast, intracellular FXIII-A activity might contribute to autophagy and apoptosis of FXIII-A expressing lymphoblast. Moreover, the product of four genes validated in our cohort, *EHMT1, ING5, MDM2, NUP43* were shown to methylate and acetylate lysyl groups of intranuclear and intracellular proteins, among others, p53, and that way they can regulate survival of neoplastic cells (25).

In addition, we showed that gene expression pattern of the FXIII-A negative subgroup overlapped with the gene expression profile of the “B-other” genetic subgroup. However, our results indicated that the FXIII-A expression status was a more powerful determinant of gene expression profile than the “B-other” status.

Fourteen genes were validated according to the FXIII-A expression status. Importantly, we were able to detect the expression of *F13A1* gene in each individual sample, and the intensity of *F13A1* expression increased in parallel with the increasing protein expression of FXIII-A among the three subgroups. Of the 14 genes, *ANGPTL2, EHMT1 FOXO1, HAP1, NUCKS1, PIK3CG, RAPGEF5, SEMA6A, SPIN1, TRH*, and *WASF2* have biologically and clinically relevant functions in GO terms, and appear to have a role in leukemia and other forms of cancer (Table 3). *NUP43* has not yet been shown to be associated with any forms of human cancer in contrast to other members of the *NUP* gene family (40). *PLAC8* which is a trophoblast lineage marker physiologically, was most intensively expressed in the FXIII-A dim subgroup. This gene has been shown to be aberrantly activated in various types of cancer arising in mammals and mammalian cancer cell lines, but not in any subtype of human ALL (24, 25). Based on *in silico* investigations we were not able to reveal a direct link between DE genes as related to the three different FXIII-A expression groups and the regulation of *F13A1* gene. Common enhancer elements of the validated DE genes and the *F13A1* make likely that common transcription factors may regulate the expression of these genes in a similar fashion.

Validated DE genes of the “B-other” subgroup overlapping with the gene expression profile of the FXIII-A negative subgroup suggested the definition of a different sub-population from the *BCR-ABL1*-like/Ph-like B-ALL subgroup discovered originally by oligonucleotide microarrays (4, 5). Evaluating the microarray results, we identified 14 DE genes in our “B-other” subgroup (*ANXA5, ARL4C, CD34, IFNGR1, MAGT1, MAPKBP1, MAP3K2, ME3, OSBPL8, PAPOLA, PTPRC, SUZ12, TACC1, TEMPO*) overlapping with DE genes in the “B-other” subgroup defined by Den Boer et al. (5). However, all the DE genes within our “B-other” group differed from class-defining genes of the so called “novel” ALL subtype introduced by Yeoh et al. (3) implying that they specify a different subtype. Each validated gene in this category, i.e., *CD3G, DFFA, GIGYF1, GIGYF2*, and *INTS3* were shown to play a role in neoplastic processes, including leukemia (41–45). However, none of these genes were published previously to be associated with pediatric BCP-ALL.

The small sample size and the low number of validated genes due to restricted budget represent an important limitation of the present study. The small number of patients involved in this investigation would make a clinical outcome analysis unreliable, even if statistically significant. Nevertheless, there was a substantially larger ratio of children who died due to their disease in the FXIII-A negative group: 5/14 children (36%), than in the FXIII-A dim group: 5/21 (24%) and in the FXIII-A bright group: 1/7 (14%). To investigate the clinical significance of FXIII-A expression in children with BCP-ALL we have started a pilot study of the ongoing BFM ALL-IC 2009 clinical trial with the participation of the Hungarian, Polish and Slovak national study groups. Preliminary analysis of this pilot study confirmed the unfavorable outcome of patients with FXIII-A negative BCP-ALL (results to be published upon completion of the pilot study). Similarly, an exact relationship between gene expression signature of FXIII-A negative samples and “B-other” samples, including *BCR-ABL1*-like/Ph-like signature, can be defined by investigating a larger number of samples from children with BCP-ALL.

In conclusion, we were the first to demonstrate the general expression of *F13A1* gene in pediatric BCP-ALL samples. The intensity of *F13A1* expression corresponded to the expression of FXIII-A protein, determined by FC in these samples. Three well-defined categories of FXIII-A protein expression: FXIII-A negative, FXIII-A dim, and FXIII-A bright subgroups defined characteristic and distinct gene expression signatures detected by Affymetrix oligonucleotide microarrays. Gene expression signature of the FXIII-A negative subgroup showed an overlap with that of the subgroup with “B-other” genetics. Validated genes proved biologically and clinically relevant. We described differential expression of genes not shown previously to be associated with pediatric BCP-ALL. Protein products of the newly identified genes may offer therapeutic targets for precision treatment of BCP-ALL. Multiparameter FC appears to be an easy-to-use and affordable method to assist in selecting pediatric patients with FXIII-A negative BCP-ALL who require a more elaborate and expensive molecular genetic investigation to design individualized therapeutic protocols.

## Data Availability Statement

The datasets generated for this study can be found in the NCBI's Gene Expression Omnibus and are accessible through GEO Series accession number GSE134480 (https://www.ncbi.nlm.nih.gov/geo/query/acc.cgi?acc=GSE134480).

## Ethics Statement

The studies involving human participants were reviewed and approved by Scientific Research Ethical Committee (TUKEB) of the Medical Research Council of Hungary: No. 43033-1/2014/EUK(423/2014). Written informed consent to participate in this study was provided by the participants' legal guardian/next of kin.

## Author Contributions

CK: conceptualization, funding acquisition, project administration, and supervision. KG, BK, SP, and GZ: data curation. KG, BK, AU, ZH, GZ, and CK: formal analysis. KG, BK, GB, PJ, AU, and GP-M: investigation. CK, AU, ZH, BS, JK, and GZ: methodology. KG, AU, JK, and GZ: resources. BK, ZH, SP, JK, and GZ: software. ZH, JK, GZ, and CK: validation. KG, BK, and GZ: visualization. GZ and CK: writing—original draft. KG, BK, AU, GB, PJ, GP-M, SP, BS, JK, GZ, and CK: writing—reviewer and editing.

### Conflict of Interest

The authors declare that the research was conducted in the absence of any commercial or financial relationships that could be construed as a potential conflict of interest.

## References

[1] Campo E Swerdlow SH Harris NL Pileri S Stein H Jaffe ES. The 2008 WHO classification of lymphoid neoplasms and beyond: evolving concepts and practical applications. Blood. (2011) 117:5019–32. 10.1182/blood-2011-01-293050 21300984 PMC3109529

[2] Arber DA Orazi A Hasserjian R Thiele J Borowitz MJ Le Beau MM. The 2016 revision to the World Health Organization classification of myeloid neoplasms and acute leukemia. Blood. (2016) 127:2391–405. 10.1182/blood-2016-03-643544 27069254

[3] Yeoh EJ Ross ME Shurtleff SA Williams WK Patel D Mahfouz R. Classification, subtype discovery, and prediction of outcome in pediatric acute lymphoblastic leukemia by gene expression profiling. Cancer Cell. (2002) 1:133–43. 10.1016/S1535-6108(02)00032-6 12086872

[4] Mullighan CG Su X Zhang J Radtke I Phillips LAA Miller CB. Deletion of IKZF1 and prognosis in acute lymphoblastic leukemia. N Engl J Med. (2009) 360:470–80. 10.1056/NEJMc090454 19129520 PMC2674612

[5] Den Boer ML van Slegtenhorst M De Menezes RX Cheok MH Buijs-Gladdines JG Peters ST. A subtype of childhood acute lymphoblastic leukaemia with poor treatment outcome: a genome-wide classification study. Lancet Oncol. (2009) 10:125–34. 10.1016/S1470-2045(08)70339-5 19138562 PMC2707020

[6] Moorman AV. New and emerging prognostic and predictive genetic biomarkers in B-cell precursor acutelymphoblastic leukemia. Haematologica. (2016) 101:407–16. 10.3324/haematol.2015.141101 27033238 PMC5004393

[7] Li JF Dai YT Lilljebjörn H Shen SH Cui BW Bai L. Transcriptional landscape of B cell precursor acute lymphoblastic leukemia based on an international study of 1,223 cases. Proc Natl Acad Sci USA. (2018) 115:E11711–20. 10.1073/pnas.1814397115 30487223 PMC6294900

[8] Kiss F Hevessy Z Veszprémi A Katona E Kiss C Vereb G. Leukemic lymphoblasts, a novel expression site of coagulation factor XIII subunit A. Thromb Haemost. (2006) 96:176–82. 10.1160/TH06-05-0270 16894461

[9] Kárai B Hevessy Z Szánthó E Csáthy L Ujfalusi A Gyurina K. Expression of coagulation factor XIII subunit A correlates with outcome in childhood acute lymphoblastic leukemia. Pathol Oncol Res. (2018) 24:345–52. 10.1007/s12253-017-0236-0 28523449

[10] Katona EE Ajzner E Tóth K Kárpáti L Muszbek L Enzyme-linked immunosorbent assay for the determination of blood coagulation factor XIIIA subunit in plasma and in cell lysates. J Immunol Methods. (2001) 258:127–35. 10.1016/S0022-1759(01)00479-3 11684129

[11] Inaba H Greaves M Mullighan CG. Acute lymphoblastic leukaemia. Lancet. (2013) 381:1943–55. 10.1016/S0140-6736(12)62187-4 23523389 PMC3816716

[12] Szklarczyk D Gable AL Lyon D Junge A Wyder S Huerta-Cepas J. STRING v11: protein-protein association networks with increased coverage, supporting functional discovery in genome-wide experimental datasets. Nucleic Acids Res. (2019) 47:D607–13. 10.1093/nar/gky1131 30476243 PMC6323986

[13] Fishilevich S Nudel R Rappaport N Hadar R Plaschkes I Iny Stein T. GeneHancer: genome-wide integration of enhancers and target genes in GeneCards. Database. (2017) 2017:1–17. 10.1093/database/bax028 28605766 PMC5467550

[14] Kim HY. Statistical notes for clinical researchers: A one-way repeated measures ANOVA for data with repeated observations. Restor Dent Endod. (2015) 40:91–5. 10.5395/rde.2015.40.1.91 25671219 PMC4320283

[15] Neveu B Spinella JF Richer C Lagacé K Cassart P Lajoie M. CLIC5: a novel ETV6 target gene in childhood acute lymphoblastic leukemia. Haematologica. (2016) 101:1534–43. 10.3324/haematol.2016.149740 27540136 PMC5479611

[16] Poulard C Kim HN Fang M Kruth K Gagnieux C Gerke DS. Relapse-associated AURKB blunts the glucocorticoid sensitivity of B cell acute lymphoblastic leukemia. Proc Natl Acad Sci USA. (2019) 116:3052–061. 10.1073/pnas.1816254116 30733284 PMC6386662

[17] Wang F Demir S Gehringer F Osswald CD Seyfried F Enzenmüller S. Tight regulation of FOXO1 is essential for maintenance of B-cell precursor acute lymphoblastic leukemia. Blood. (2018) 131:2929–42. 10.1182/blood-2017-10-813576 29622548

[18] Han J Jin R Zhang M Guo Q Zhou F. Ikaros 6 protects acute lymphoblastic leukemia cells against daunorubicin-induced apoptosis by activating the Akt-FoxO1 pathway. J Leukoc Biol. (2017) 101:675–81. 10.1189/jlb.2A0116-040RR 27707884

[19] Köhrer S Havranek O Seyfried F Hurtz C Coffey GP Kim E. Pre-BCR signaling in precursor B-cell acute lymphoblastic leukemia regulates PI3K/AKT, FOXO1and MYC, and can be targeted by SYK inhibition. Leukemia. (2016) 30:1246–54. 10.1038/leu.2016.9 26847027 PMC5459356

[20] Lee JK Kang S Wang X Rosales JL Gao X Byun HG. HAP1 loss confers l-asparaginase resistance in ALL by downregulating the calpain-1-Bid-caspase-3/12 pathway. Blood. (2019) 133:2222–32. 10.1182/blood-2018-12-890236 30819925 PMC6587669

[21] Akbari Moqadam F Lange-Turenhout EA Ariës IM Pieters R den Boer ML. MiR-125b, miR-100 and miR-99a co-regulate vincristine resistance in childhood acute lymphoblastic leukemia. Leuk Res. (2013) 37:1315–21. 10.1016/j.leukres.2013.06.027 23915977

[22] Sanchez VE Nichols C Kim HN Gang EJ Kim YM. Targeting PI3K signaling in acute lymphoblastic leukemia. Int J Mol Sci. (2019) 20:E412. 10.3390/ijms20020412 30669372 PMC6358886

[23] Archer MC. Role of sp transcription factors in the regulation of cancer cell metabolism. Genes Cancer. (2011) 2:712–9. 10.1177/1947601911423029 22207896 PMC3218407

[24] Grate LR. Many accurate small-discriminatory feature subsetsexist in microarray transcript data: biomarker discovery. BMC Bioinformatics. (2005) 6:97. 10.1186/1471-2105-6-97 15826317 PMC1090559

[25] Cabreira-Cagliari C Dias NC Bohn B Fagundes DGDS Margis-Pinheiro M Bodanese-Zanettini MH. Revising the PLAC8 gene family: from a central role in differentiation, proliferation, and apoptosis in mammals to a multifunctional role in plants. Genome. (2018) 61:857–65. 10.1139/gen-2018-0035 30427722

[26] Liu W Zhao J Jin M Zhou M circRAPGEF5 contributes to papillary thyroid proliferation and metastasis by regulation miR-198/FGFR1. Mol Ther Nucleic Acids. (2019) 14:609–16. 10.1016/j.omtn.2019.01.003 30785065 PMC6379567

[27] Gandemer V Rio AG de Tayrac M Sibut V Mottier S Ly Sunnaram B. Five distinct biological processes and 14 differentially expressed genes characterize TEL/AML1-positive leukemia. BMC Genomics. (2007) 8:385. 10.1186/1471-2164-8-385 17956600 PMC2211320

[28] Fang Z Cao B Liao JM Deng J Plummer KD Liao P. SPIN1 promotes tumorigenesis by blocking the uL18 (universal large ribosomal subunit protein 18)-MDM2-p53 pathway in human cancer. ELife Sci. (2018) 7:e31275. 10.7554/eLife.31275 29547122 PMC5871334

[29] Frölich E Wahl R The forgotten effects of thyreotropin-releasing hormone: metabolic functions and medical applications. Front Neuroendocrinol. (2019) 52:29–43. 10.1016/j.yfrne.2018.06.006 29935915

[30] Stransky N Cerami E Schalm S Kim JL Lengauer C. The landscape of kinase fusions in cancer. Nat Commun. 5:4846. 10.1038/ncomms5846 25204415 PMC4175590

[31] Invernizzi R De Fazio P Iannone AM Zambelli LM Rastaldi MP Ippoliti G. Immunocytochemical detection of fac- tor XIII A–subunit in acute leukemia. Leuk Res. (1992) 16:829–36. 10.1016/0145-2126(92)90163-2 1382173

[32] Kappelmayer J Simon A Katona E Szanto A Nagy L Kiss A. Coagulation factor XIII-A. A flow cytometric intracellular marker in the classification of acute myeloid leukemias. Thromb Haemost. (2005) 94:454–9. 10.1160/TH05-03-0206 16113839

[33] Kiss F Simon A Csáthy L Hevessy Z Katona E Kiss C. A coagulation factor becomes useful in the study of acute leukemias: studies with blood coagulation factor XIII. Cytometry A. (2008) 73:194–201. 10.1002/cyto.a.20485 18000871

[34] Kida M Souri M Yamamoto M Saito H Ichinose A. Transcriptional regulation of cell type-specific expression of the TATA-less A subunit gene for human coagulation factor XIII. J Biol Chem. (1999) 274:6138–47. 10.1074/jbc.274.10.6138 10037697

[35] Gratchev A Kzhyshkowska J Utikal J Goerdt S. Interleukin-4 and dexamethasone counterregulate extracellular matrix remodelling and phagocytosis in type-2 macrophages. Scand J Immunol. (2005) 61:10–7. 10.1111/j.0300-9475.2005.01524.x 15644118

[36] ENCODE Project Consortium A user's guide to the encyclopedia of DNA elements (ENCODE). PLoS Biol. (2011) 9:e1001046 10.1371/journal.pbio.1001046 21526222 PMC3079585

[37] Mitchell JL Mutch NJ. Let's cross-link: diverse functions of the promiscuous cellular transglutaminase factor XIII-A. J Thromb Haemost. (2019) 17:19–30. 10.1111/jth.14348 30489000

[38] Richardson VR Cordell P Standeven KF Carter AM Substrates of factor XIII-A: roles in thrombosis and wound healing. Clin Sci. (2013) 3:123–37. 10.1042/CS20120233 23075332

[39] Huang J Dorsey J Chuikov S Pérez-Burgos L Zhang X Jenuwein T. G9a and Glp methylate lysine 373 in the tumor suppressor p53. J of Biol Chem. (2010) 285:9636–41. 10.1074/jbc.M109.062588 20118233 PMC2843213

[40] Nofrini V Di Giacomo D Mecucci C Nucleoporin genes in human disease. Eur J Human Genet. (2016) 24:1388–95. 10.1038/ejhg.2016.25 27071718 PMC5027676

[41] Zamani-Ahmadmahmudi M Najafi A Nassiri SM. Detection of critical genes associated with Overall Survival (OS) and Progression-Free Survival (PFS) in reconstructed canine B-cell lymphoma Gene Regulatory Network (GRN). Cancer Invest. (2016) 34:70–9. 10.3109/07357907.2015.1114120 26818715

[42] Yi B Zhang M Schwartz-Albiez R Cao Y. Mechanisms of the apoptosis induced by CD176 antibody in human leukemic cells. Int J Oncol. (2011) 38:1565–73. 10.3892/ijo.2011.992 21455576

[43] Ajiro M Nishidate T Katagiri T Nakamura Y. Critical involvement of RQCD1 in the EGFR-Akt pathway in mammary carcinogenesis. Int J Oncol. (2010) 37:1085–93. 10.3892/ijo_00000760 20878056

[44] Christen F Hoyer K Yoshida K Hou HA Waldhueter N Heuser M. Genomic landscape and clonal evolution of acute myeloid leukemia with t(8;21): an international study on 331 patients. Blood. (2019) 133:1140–51. 10.1182/blood-2018-05-852822 30610028

[45] Federico A Rienzo M Abbondanza C Costa V Ciccodicola A Casamassimi A. Pan-cancer mutational and transcriptional analysis of the integrator complex. Int J Mol Sci. (2017) 18:E936. 10.3390/ijms18050936 28468258 PMC5454849

