# Supplementary material for: Coagulation FXIII-A Protein Expression Defines Three Novel Sub-populations in Pediatric B-Cell Progenitor Acute Lymphoblastic Leukemia Characterized by Distinct Gene Expression Signatures
Source: Front Oncol. 2019 Oct 25;9:1063. doi: 10.3389/fonc.2019.01063 (PMC6823876; doi:10.3389/fonc.2019.01063)
Supplement: Supplementary file 1 [file Table_1.docx]

**Supplementary Table 1.** Over-represented GO biological processes having the lowest corrected p-values in the FXIII-A status comparison

| GO ID | GO annotation | Corrected p-value |
| --- | --- | --- |
| GO:0016569 | covalent chromatin modification | 3,20E-05 |
| GO:0016570 | histone modification | 3,59E-05 |
| GO:0006325 | chromatin organization | 1,07E-04 |
| GO:0050779 | RNA destabilization | 7,55E-04 |
| GO:1900118 | negative regulation of execution phase of apoptosis | 1,14E-03 |
| GO:0034248 | regulation of cellular amide metabolic process | 1,29E-03 |
| GO:0010608 | posttranscriptional regulation of gene expression | 1,93E-03 |
| GO:0010608 | posttranscriptional regulation of gene expression | 1,93E-03 |
| GO:0006417 | regulation of translation | 1,97E-03 |
| GO:0097284 | hepatocyte apoptotic process | 1,99E-03 |
| GO:0061038 | uterus morphogenesis | 2,07E-03 |
| GO:0071478 | cellular response to radiation | 2,10E-03 |
| GO:0061157 | mRNA destabilization | 2,35E-03 |
| GO:0031330 | negative regulation of cellular catabolic process | 2,52E-03 |
| GO:0071482 | cellular response to light stimulus | 2,76E-03 |
| GO:0016571 | histone methylation | 3,34E-03 |
| GO:0033169 | histone H3-K9 demethylation | 3,59E-03 |
| GO:0104004 | cellular response to environmental stimulus | 4,76E-03 |
| GO:0071214 | cellular response to abiotic stimulus | 4,76E-03 |
| GO:2000767 | positive regulation of cytoplasmic translation | 5,63E-03 |
| GO:0048048 | embryonic eye morphogenesis | 5,65E-03 |
| GO:0032074 | negative regulation of nuclease activity | 6,76E-03 |
| GO:0043487 | regulation of RNA stability | 7,30E-03 |
| GO:0009895 | negative regulation of catabolic process | 7,49E-03 |
| GO:0048596 | embryonic camera-type eye morphogenesis | 9,13E-03 |
| GO:0018205 | peptidyl-lysine modification | 9,39E-03 |
