# Supplementary material for: Coagulation FXIII-A Protein Expression Defines Three Novel Sub-populations in Pediatric B-Cell Progenitor Acute Lymphoblastic Leukemia Characterized by Distinct Gene Expression Signatures
Source: Front Oncol. 2019 Oct 25;9:1063. doi: 10.3389/fonc.2019.01063 (PMC6823876; doi:10.3389/fonc.2019.01063)
Supplement: Supplementary file 2 [file Table_2.docx]

| GO ID | GO annotation | Corrected p-value |
| --- | --- | --- |
| GO:0070228 | regulation of lymphocyte apoptotic process | 9,96E-04 |
| GO:0070229 | negative regulation of lymphocyte apoptotic process | 4,18E-03 |
| GO:0070232 | regulation of T cell apoptotic process | 4,76E-03 |
| GO:2001238 | positive regulation of extrinsic apoptotic signaling pathway | 3,46E-03 |
| GO:0009409 | response to cold | 1,91E-03 |
| GO:0043124 | negative regulation of I-kappaB kinase/NF-kappaB signaling | 3,43E-03 |
| GO:1905517 | macrophage migration | 2,15E-03 |
| GO:0048246 | macrophage chemotaxis | 4,73E-03 |
| GO:1905521 | regulation of macrophage migration | 3,13E-03 |
| GO:0007223 | Wnt signaling pathway, calcium modulating pathway | 4,72E-03 |
| GO:0070227 | lymphocyte apoptotic process | 2,21E-03 |
| GO:2000106 | regulation of leukocyte apoptotic process | 3,06E-03 |
| GO:0070231 | T cell apoptotic process | 2,87E-03 |
| GO:2000107 | negative regulation of leukocyte apoptotic process | 1,01E-02 |
| GO:0006968 | cellular defense response | 1,32E-02 |

**Supplementary Table 2.** Over-represented GO biological processes in the ’B-other’ status comparison
