# Supplementary material for: Coagulation FXIII-A Protein Expression Defines Three Novel Sub-populations in Pediatric B-Cell Progenitor Acute Lymphoblastic Leukemia Characterized by Distinct Gene Expression Signatures
Source: Front Oncol. 2019 Oct 25;9:1063. doi: 10.3389/fonc.2019.01063 (PMC6823876; doi:10.3389/fonc.2019.01063)
Supplement: Supplementary file 3 [file Table_3.docx]

|  | Microarray fold-changes | | | RT-Q-PCR fold-changes | | |
| --- | --- | --- | --- | --- | --- | --- |
|  | **bright/neg** | **bright/dim** | **neg/dim** | **bright/neg** | **bright/dim** | **neg/dim** |
| **ANGPTL2** | 4.42 | 3.38 | 0.76 | 16.94 | 2.89 | 0.17 |
| **EHMT1** | 1.45 | 2.45 | 1.69 | 1.86 | 1.60 | 0.86 |
| **F13A1** | 5.45 | 3.39 | 0.62 | 7.20 | 2.50 | 0.35 |
| **FOXO1** | 1.46 | 2.70 | 1.85 | 3.84 | 5.07 | 1.32 |
| **HAP1** | 1.58 | 1.50 | 0.95 | 17.71 | 3.41 | 0.19 |
| **NUCKS1** | 1.33 | 2.50 | 1.88 | 1.58 | 1.62 | 1.03 |
| **NUP43** | 1.45 | 1.97 | 1.36 | 1.43 | 1.26 | 0.88 |
| **PIK3CG** | 1.47 | 1.85 | 1.26 | 1.60 | 1.48 | 0.92 |
| **PLAC8*** |  |  |  | 0.69 | 0.46 | 0.67 |
| **RAPGEF5** | 5.23 | 2.99 | 0.57 | 5.90 | 2.55 | 0.43 |
| **SEMA6A** | 2.52 | 2.84 | 1.13 | 4.60 | 2.67 | 0.58 |
| **SPIN1** | 1.68 | 2.59 | 1.54 | 2.05 | 1.64 | 0.80 |
| **TRH** | 7.51 | 3.23 | 0.43 | 12.64 | 3.37 | 0.27 |
| **WASF2** | 2.49 | 3.30 | 1.32 | 2.65 | 1.90 | 0.72 |

**Supplementary Table 3.** Comparison of fold-changes of validated DE genes according to FXIII-A expression subgroups by Affymetrix MicroArray and RT-Q-PCR

*PLAC8 was not found significant by microarray measurements.
